# The influence of pneumococcal conjugate vaccine-13 on nasal colonisation in a controlled human infection model of pneumococcal carriage in Malawi: a double-blinded randomised controlled trial protocol

**DOI:** 10.12688/wellcomeopenres.17172.2

**Published:** 2022-06-16

**Authors:** Ben Morton, Kondwani Jambo, Tarsizio Chikaonda, Jamie Rylance, Marc Y.R. Henrion, Ndaziona Peter Banda, Edna Nsomba, Joel Gondwe, Daniela Ferreira, Stephen B. Gordon, MARVELS Consortium

**Affiliations:** 1 Lung Health, Malawi-Liverpool Wellcome Trust Clinical Research Programme, Blantyre, Malawi; 2 Department of Clinical Sciences, Liverpool School of Tropical Medicine, Liverpool, UK; 3 Critical Care Medicine, Liverpool University Hospitals NHS Foundation Trust, Liverpool, UK; 4 Department of Medicine, Queen Elizabeth Central Hospital, Blantyre, Malawi; 5 College of Medicine, Malawi, Blantyre, Malawi

**Keywords:** Streptococcus pneumoniae, Pneumococcal carriage, Experimental medicine, pneumonia, vaccine, controlled human infection model, nasal colonisation, global health

## Abstract

*Streptococcus pneumoniae* is the leading cause of morbidity and mortality due to community acquired pneumonia, bacterial meningitis and bacteraemia worldwide. Pneumococcal conjugate vaccines protect against invasive disease, but are expensive to manufacture, limited in serotype coverage, associated with serotype replacement, and demonstrate reduced effectiveness against mucosal colonisation.  For Malawi, nasopharyngeal carriage of vaccine-type pneumococci is common in vaccinated children despite national roll-out of 13-valent pneumococcal conjugate vaccine (PCV13) since 2011. Our team has safely transferred an established experimental human pneumococcal carriage method from Liverpool School of Tropical Medicine to the Malawi-Liverpool Wellcome Trust Clinical Research Programme, Malawi.

This study will determine potential immunological mechanisms for the differential effects of PCV13 on nasal carriage between healthy Malawian and UK populations. We will conduct a double-blinded randomised controlled trial to vaccinate (1:1) participants with either PCV13 or control (normal saline). After a period of one month, participants will be inoculated with *S. pneumoniae *serotype 6B to experimentally induce nasal carriage using the EHPC method. Subsequently, participants will be invited for a second inoculation after one year to determine longer-term vaccine-induced immunological effects. Primary endpoint: detection of inoculated pneumococci by classical culture from nasal wash recovered from the participants after pneumococcal challenge. Secondary endpoints: local and systemic innate, humoral and cellular responses to PCV-13 with and without pneumococcal nasal carriage

The primary objective of this controlled human infection model study is to determine if PCV-13 vaccination is protective against pneumococcal carriage in healthy adult Malawian volunteers. This study will help us to understand the observed differences in PCV-13 efficacy between populations and inform the design of future vaccines relevant to the Malawian population.

**Trial Registration:** Pan African Clinical Trial Registry (REF: PACTR202008503507113)

## Abbreviations

AE: Adverse Event; CAP: Community Acquired Pneumonia; CFU: Colony Forming Units; CHIM: Controlled Human Infection Model; CRF: Case Report Form; DSMB: Data, Safety and Monitoring Board; EHPC: Experimental Human Pneumococcal Colonisation; ELISA: Enzyme-Linked Immunosorbent Assay; ELISPOT: Enzyme-Linked Immune Absorbent Spot; FBC: Full Blood Count; GCP: Good Clinical Practice; HIV: Human Immunodeficiency Virus; HTS: Human immunodeficiency virus Testing Service; ICH-GCP: International Conference on Harmonisation of Good Clinical Practice; LSTM: Liverpool School of Tropical Medicine; MARVELS: Malawi Accelerated Research in Vaccines, Experimental and Laboratory Systems; MHRA; Medicines and Healthcare products Regulatory Authority (UK Regulator); MK: Malawian Kwacha; MLW: Malawi-Liverpool Wellcome Trust Clinical Research Programme Laboratories; MTA: Material Transfer Agreement; NHSRC: National Health Sciences Research Committee; OM: Otitis Media; PBMC: Peripheral Blood Mononuclear Cell; PCR: Polymerase Chain Reaction; PCV: Pneumococcal conjugate vaccines; PCV13: Pneumococcal conjugate vaccine 13; PCVPA: Pneumococcal Carriage in Vulnerable Populations in Africa; PMPB: Pharmacy, Medicines and Poisons Board; QECH: Queen Elizabeth Central Hospital; RNA: Ribonucleic Acid; SAE: Serious Adverse Event; SOP: Standardised Operating Procedure; SUSAR: Serious Unexpected Serious Adverse Reaction; TSC: Trial Steering Committee.

## Introduction

*Streptococcus pneumoniae* remains a major public health threat worldwide, particularly in low- to middle-income countries ^1^. Despite the introduction of pneumococcal conjugate vaccines (PCV), there remain more than 500,000 pneumococcal deaths in children 0–59 months old per year ^2^. The introduction PCVs coincided with a 51% reduction in pneumococcal deaths between 2000 and 2015 but these remain expensive to manufacture, have limited serotype coverage, with evidence of serotype replacement and have reduced efficacy against mucosal colonisation (13–20%) compared to invasive disease ^3^. In Malawi, nasopharyngeal carriage of vaccine-type pneumococci is common in vaccinated children despite national roll-out of 13-valent pneumococcal conjugate vaccine (PCV13) since 2011 ^4^. Cost-effectiveness estimates for PCV-13 vaccination are based strongly on improved herd immunity through reduced carriage (reservoir for infection transmission) in vaccinated children ^5^.

We established a safe and reproducible Controlled Human Infection Model (CHIM) at the Liverpool School of Tropical Medicine (LSTM), UK. This model has been used to test the effect of PCV-13 versus control (Hepatitis A) vaccination in a Phase III trial ^6^. Importantly, we found that PCV-13 induces protection against controlled infection model induced carriage in the UK ^6^. This finding was entirely consistent with the observed impact of PCV-13 on nasal carriage at the UK and US population level. Most recently, we have successfully transferred operating procedures from the LSTM model and safely adapted these for the Malawian context (National Health Sciences Research Committee [NHSRC]: 19/08/2246) ^7^.

Our feasibility study of pneumococcal controlled human infection in Malawi ^7^ has demonstrated no SAEs or AEs and minimal symptoms for participants who completed the programme ^8^. We have confirmed LSTM procedures and successfully adapted standard operating procedures for in-country use. Our observed nasal carriage rates after inoculation of 3/9 (33%) and 4/9 at the 20,000 and 80,000 CFU doses (S. pneumoniae serotype 6B BHN418), respectively. The 4/9 carriage rate at 80,000 CFUs met our predefined study stopping criteria ^4^ to confirm CHIM feasibility. This confirms that the 80,000 CFU pneumococcal dose used by LSTM to test vaccines in the UK is relevant to populations within Malawi ^8^. Based on successful transfer of procedures, we are confident that our clinical and laboratory procedures are sufficiently robust to test vaccine efficacy. Our robust social science led exploration of acceptability has demonstrated that participants feel safe, cared for and were proud to be involved with this research programme ^9^.

This study will initially confirm if the epidemiological observation of vaccine-type pneumococcal carriage in vaccinated children in Malawi is mirrored by a diminished impact of PCV13 in reducing nasopharyngeal carriage in healthy adults in Malawi compared to the UK. It is likely that this will be the case as the intensity of community exposure, environmental conditions and host immunological history are very different in Malawi compared to the UK. It is possible, however, that the adult model will not show this reduced efficacy in which case we will have to refine our study population to more accurately reflect the populations of interest. In the event that reduced vaccine efficacy is seen in the Malawi CHIM model, we will then determine potential immunological mechanisms for the differential effects of PCV-13 on nasal carriage between healthy Malawian and UK populations.

The burden of both nasal carriage rates ^10^ and invasive infection ^2^ are much higher in Malawian compared to UK populations. As above, increased background exposure to pneumococcus is a potential mechanism for reduced vaccine efficacy ^11^. Therefore, we will test vaccine effectiveness against a sequentially increased pneumococcal exposure dose of 20,000, 80,000 and 160,000 colony forming units per nostril. The 160,000 dose has been safely administered in Liverpool with no observed adverse events ^12^. We will use the established S. pneumoniae serotype 6B BHN418 challenge agent strain (prepared and aliquoted in LSTM laboratories and transferred to Malawi). The preparation process is subject to independent identification, typing and assessment of antimicrobial sensitivity by Public Health England. Further, PCV-13 induced serotype-specific antibody concentrations are known to wane in the months following primary vaccination, potentially becoming too low to prevent invasive disease in vulnerable groups ^13^. We will therefore invite participants to test vaccine efficacy at twelve months after vaccination with a second 80,000 cfu/nostril challenge with pneumococcus to determine longer-term immune responses in the PCV-13 vaccinated group. Previous nasal carriage is known to be protective against re-challenge ^12^. Therefore, we will also invite participants from the control vaccine group for re-challenge at 12-months to differentiate whether any continued protective effects are vaccine or carriage induced. We will directly compare data generated from this Malawian study with existing UK data ^6^ to compare immunological parameters and define the mechanisms for observed differences in vaccine-mediated effects between the two populations.

There is an urgent need to develop new vaccines directed to the Malawian population. We will determine immunological responses to PCV-13 against a relevant pneumococcal challenge (6B is a vaccine serotype) in Malawi and contrast these with existing data from a completed UK study (study design, CHIM and vaccines directly comparable). This information will help us to understand the observed differences in PCV-13 efficacy between populations and inform the design of future vaccines relevant to the Malawian population.

### Ethical considerations for controlled human infection studies in low income settings

We conducted extensive preliminary work as a precursor to the introduction of the pneumococcal controlled human infection model to Malawi. This included extensive stakeholder consultation ^14^ and detailed exploration of community knowledge and views regarding the CHIM of pneumococcal carriage in Malawi ^15^. This work demonstrated that stakeholders perceived CHIM research had potential population health benefits but that there was a need to balance equity, safety and relevance in inclusion criteria. Further, this work demonstrated that ongoing assessment of participant experience was required. Informed by this, we interviewed participants during our feasibility study to assess acceptability and determine ways to optimise their experience ^9^. This work demonstrated that participation was based on three essential themes: motivation to participate, compensation and advocacy. We have incorporated lessons learnt from structured enquiry within our feasibility study into this study design.

## Study protocol (V1.2, 16 ^th^ Dec 2020)

### Main objective

Determine if PCV-13 vaccination is protective against experimental human pneumococcal carriage (EHPC) using a CHIM in healthy adult Malawian volunteers and compare the level of protection with that observed in the same CHIM in Liverpool, UK.

### Secondary objectives

1. Determine how pneumococcal dose influences carriage in PCV-13 vaccinated adults
2. Determine PCV-13 protection against pneumococcal re-challenge 12-months post vaccination
3. Determine the protective effect of prior pneumococcal carriage in Malawian volunteers
4. Examine local and systemic innate, humoral and cellular responses to PCV-13 with and without pneumococcal nasal carriage
5. Explore participant experience in the study to monitor acceptability

### Study hypothesis

Vaccination with PCV-13 will reduce nasal carriage by 40% compared to control vaccination with normal (0.9%) saline (from baseline of 60% carriage to 36% carriage) in participants challenged with pneumococcus at a dose of 80,000 cfu/naris.

### Study design

A double-blinded randomised controlled trial of adult healthy human participants experimentally exposed to escalating doses of *S. pneumoniae* in the nasopharynx. The intervention vaccine will be PCV-13 (Prevnar-13) and the control vaccine will be normal (0.9%) saline. We will closely monitor study participants to ensure safety and tolerability of study procedures. We will measure immune protective responses to *S. pneumoniae* challenge using mucosal (nasal, throat and saliva) and blood samples. We will invite participants for re-challenge 12-months after vaccination to determine longer-term immunological responses.

This study is based on the foundation of our ethically approved feasibility study ^7– 9^ and will utilise standard operating procedures successfully validated in Malawi. The study follows a logical sequence and the detailed schedule of visits (12 scheduled visits - Visit A to Visit L) and procedures carried out at each visit are described in Table 1.

The sequence of stages followed is:

1. **Screening and recruitment:** Potential participants will be screened to ensure health and safety ( *Extended data* ^16^) as detailed in the Methods section.

2. **Randomisation:** Participants will be allocated to vaccination with PCV-13 or normal saline vaccination in randomised blocks (1:1 randomisation PCV13 vs. normal saline, double-blinded).

3. **Post vaccination samples:** Samples will be taken to confirm nasal carriage status and measure immune response.

4. **Primary inoculation:** Participants will be inoculated with *S. pneumoniae* serotype 6B to the inside of each naris. We will sequentially increase the inoculation dose in groups of patients (20,000, 80,000 and 160,000 colony forming units per naris). One hundred and forty participants will receive the 80,000 dose to facilitate direct comparisons with our completed UK study ^6^. Forty participants will receive the 20,000 dose and twenty participants will receive the 160,000 dose. Total participants = 200.

**5.** **Detection of pneumococcal carriage:** Nasal wash samples will be taken, according to a standardized protocol, at days 2, 7, and 14 days post inoculation. Classical microbiological culture will determine nasal colonisation with pneumococcal serotype 6B at each time point.

**6.** **Immunology response measurements:** Blood, mucosal and nasal cell samples will be taken to determine the immunological response to nasal challenge.

**7.** **Re-screen and recruitment**: Participants will be invited to be re-challenged with pneumococcus. We will re-screen to ensure health and safety as detailed in Methods.

**8.** **Final inoculation:** Participants will be inoculated with *S. pneumoniae* serotype 6B to the inside of each naris (as per stage four). We will use a single dose of 80,000 cfu/naris. This is the most established dose based on completed UK studies with robust colonisation data.

**9.** **Detection of pneumococcal carriage:** Nasal wash samples will be taken, according to a standardized protocol, at days 2, 7, and 14 days post inoculation (as per stage five). Classical microbiological culture will determine nasal colonisation with pneumococcal serotype 6B at each time point.

**Table 1.** Study procedures and sampling schedule. Study visit A may occur for up to one month before study visit B. There will be a flexibility of up to 10 weeks after study visit B (1–11 weeks) to allow for staggering to inoculate a safe number of participants per week. Thereafter, the schedule will fix as per the table (e.g. if study visit D occurs at week 9, then E will be at 9, F at 10 and so on). There will be a further flexibility ± two weeks at visit H to facilitate appointment booking. There will be a tolerance of ± two working days for study visits F, G, K and L. FBC: full blood count; PBMCs: peripheral blood mononuclear cells; RNA: ribonucleic acid. *This is for immune measures (transcriptomics) and is not a genetic test. Serum will be used for serological testing; inflammatory markers; and biochemistry. Pregnancy test for female participants only.

Study Visit	A	B	C	D	E	F	G	First phase of study completed. Participants invited for second phase consent one -year post vaccination	H	I	J	K	L
Study Week		1	5	6	6	7	8		53	54	54	55	56
Day post inoculation				0	2	7	14			0	2	7	14
Consent (Written)		x							x				
Consent (Verbal)	x	x	x	x	x	x	x		x	x	x	x	x
Clinical Exam		x							x				
Vital Signs		x	x	x	x	x	x		x	x	x	x	x
Medical History		x							x				
Screen for AEs		x	x	x	x	x	x		x	x	x	x	x
Randomisation		x											
Vaccination		x											x
Inoculation				x						x			
HIV test		x	x						x				
Pregnancy test		x	x						x				
FBC (2.5ml)		x							x				
PBMCs (18ml)		x	x		x	x	x		x		x	x	x
Serum (2.5ml)		x	x		x	x	x		x		x	x	x
Blood RNA (2.5ml) *		x	x		x				x		x		
Nasosorption		x	x	x	x	x	x		x	x	x	x	x
Nasal Wash		x	x		x	x	x		x		x	x	x
Nasal cells		x	x		x	x	x		x		x	x	x
Throat swab		x	x		x	x	x		x		x	x	x
Saliva		x	x		x	x	x		x		x	x	x

### Study endpoints

***Primary endpoint:*** detection of the inoculated pneumococci by classical culture methods, at any time point, from nasal wash recovered from the participants at days 2, 7 and 14 following the initial pneumococcal challenge.

***Secondary endpoints:*** Pneumococcal carriage density and duration will also be measured to inform the primary endpoint. Innate, humoral and cellular responses to pneumococcal colonisation will be assessed by immunological assays on collected samples. These data will allow us to define the host variables that predict colonisation and protection due to PCV-13 vaccination.

***Social science evaluation:*** An exit questionnaire ( *Extended data* ^17^) will be conducted as part of visit G and visit L to monitor participant satisfaction and acceptability of the study and procedures. Purposeful sampling will be conducted on a subset of individuals to explore acceptability in more detail. This work will focus on perceptions of and concerns about controlled human infection; experience of engagement activities and consent processes; and participant recommendations for future research.

### Study setting

Clinical procedures will be conducted at the Queen Elizabeth Central Hospital Research Ward, Blantyre, Malawi. Laboratory procedures will be conducted at the adjacent Malawi-Liverpool Wellcome Trust (MLW) Clinical Research Programme Laboratories.

### Recruitment target

We will recruit 200 healthy adult participants to complete the study.

### Duration

Recruitment of all participants and follow up will be completed within 28 months.

### Participants, schedule and timelines

**Figure 1.**
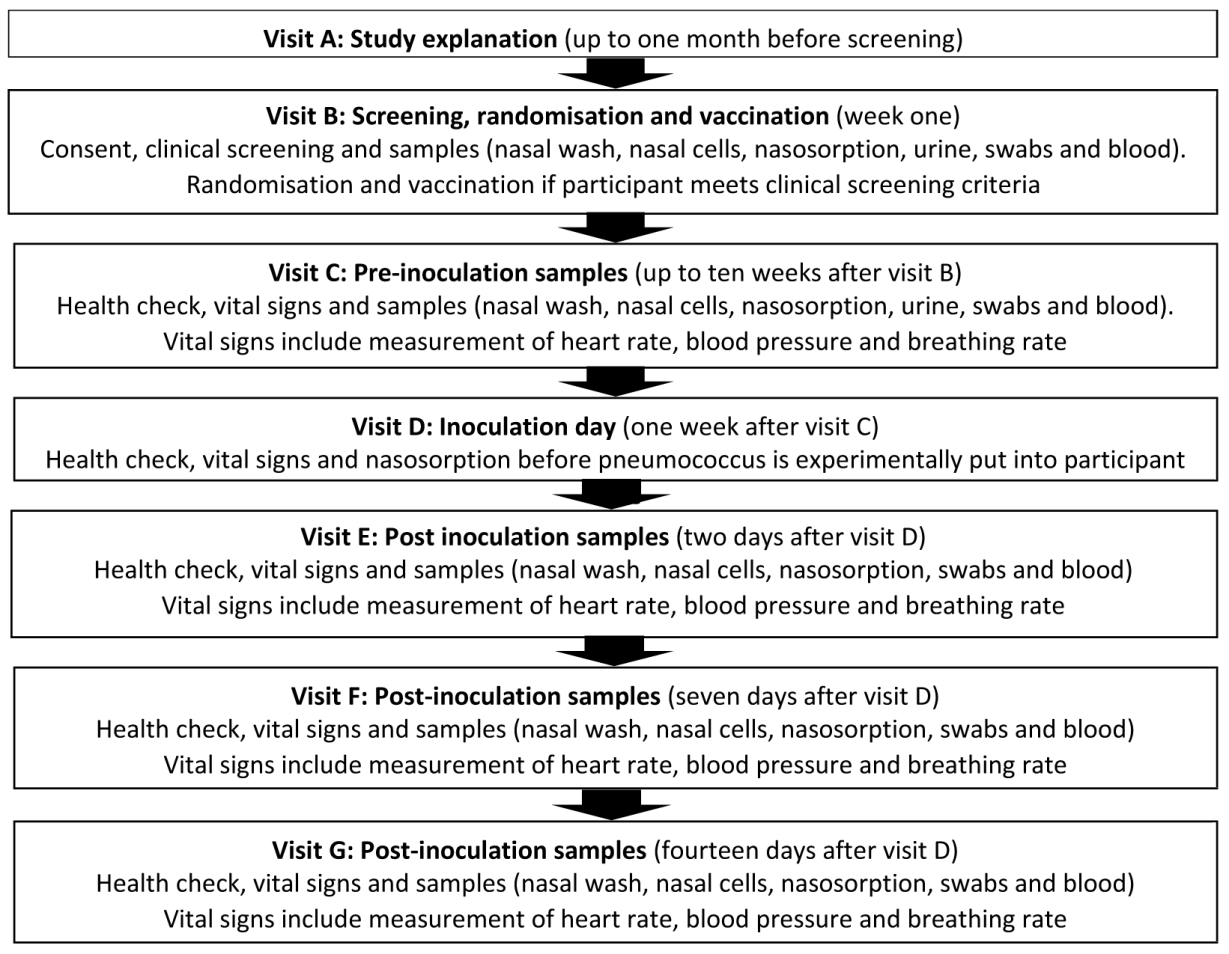
Visit schedule for Phase 1 of the study.

**Figure 2.**
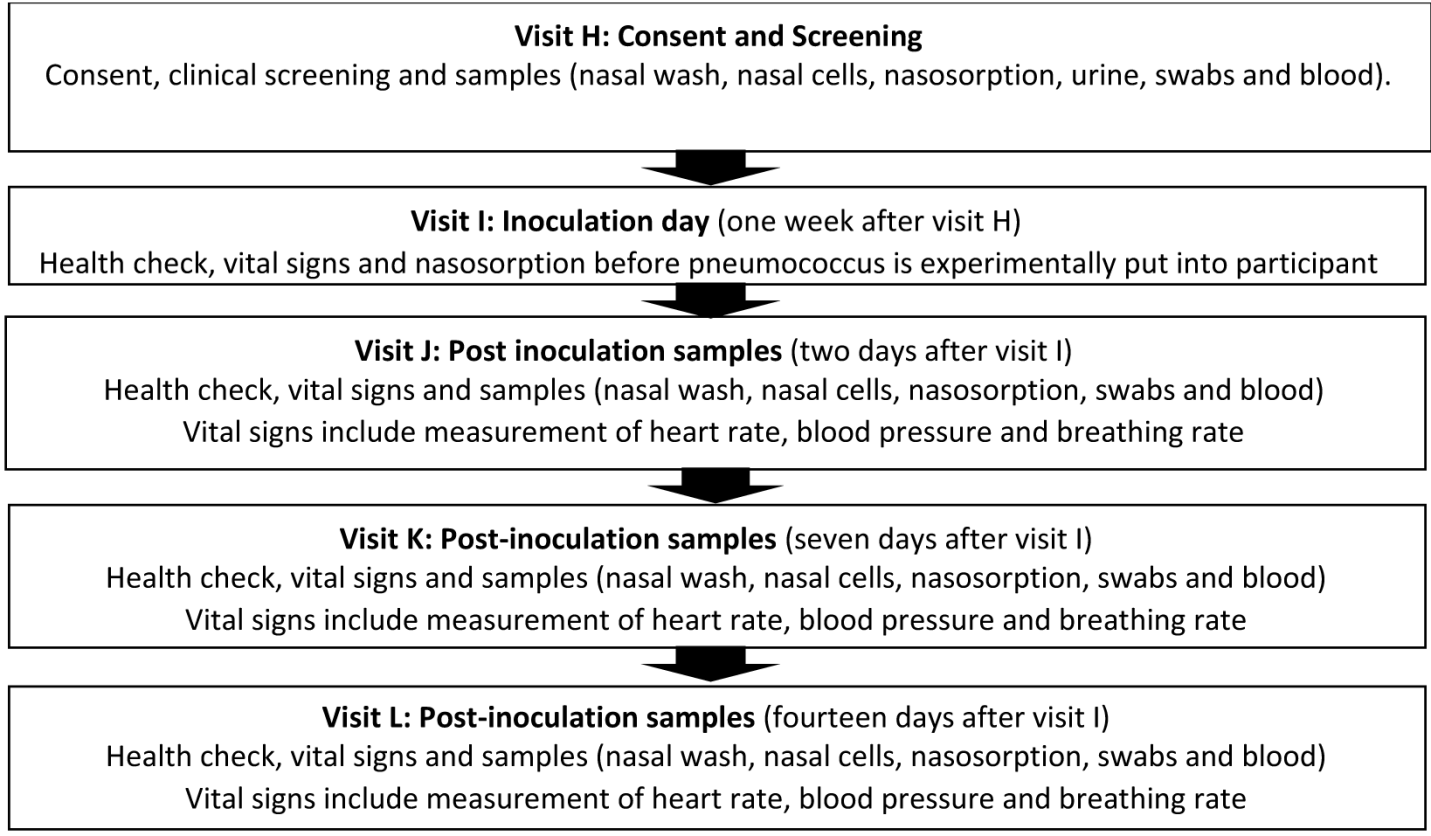
Visit schedule for Phase 2 of the study one-year post vaccination. Participation in phase 2 is not mandatory. An additional consent procedure will be required prior to participation in this phase.

We will inoculate healthy adult participants who do not smoke tobacco or other products (including electronic cigarettes) with a well-characterised, fully sequenced penicillin-sensitive pneumococci and observe them for the development of pneumococcal carriage up to 14 days post inoculation. Study discharge (with safety net advice) will occur after visit G (day 14 post inoculation – see Table 1). Participants will be invited to attend for re-recruitment leading to re-inoculation twelve months after vaccination (visit H – see Table 1). This process will include repeated participant information delivery and health screening procedures to confirm informed consent and continued health status ( *Extended data* ^16^). The visit schedule is also described in detail in Figure 1 and Figure 2.

Study visits must take place according to the proposed sequence in order to ensure participant safety. This will be clearly explained to the participants during recruitment and consent ( *Extended data* ^16^). If a participant is unable to comply, they will not be recruited. Daily phone contact occurs between study visits until day seven post-inoculation.

### Participant recruitment

Advertisements inviting healthy participants to participate will be widely placed. Areas will include physical notice boards, table display, electronic notice boards, the intranet/internet of local universities and colleges (with permission), social media, the local press, television and radio. Staff from the MLW team will be permitted to participate providing they are study team members.

Interested persons will be asked to contact the research team by text for further information. Once potentially interested participants have contacted the research team, they will then be invited to meet a member of the research team (information dissemination – visit A, Table 1). A research team member will explain the study at the meeting and the Participant Information Sheet will be provided. Participants will then consider if they wish to continue (see Table 1).

At visit B, the informed consent process will be conducted by either a research nurse or research doctors ( *Extended data* ^16^) who are formally delegated by the Chief Investigator, trained in good clinical practice (GCP), consent and the trial protocol. Following consent, inclusion and exclusion criteria will be applied as below.

***Inclusion criteria***

• Adults aged 18–40 years - ages chosen to minimise the risk of pneumococcal infection
• Fluent spoken and written Chichewa or English - to ensure a comprehensive understanding of the research project, their proposed involvement and communication with all members of the research team

***Exclusion criteria***

• Previous pneumococcal vaccination
• SARS-CoV-2 RT-qPCR (Polymerase Chain Reaction) positive at screening or any subsequent visit (throat swab)
• HIV-infection seropositive. HIV self-test kits will be used by participants to confirm infection status in the presence of the research team. Any seropositive participant will be referred to trained personnel certified in HIV Testing Services (HTS) for confirmatory “Determine” and “Unigold” tests. Any participants confirmed as HIV-infection positive will be referred to the governmental system for infection confirmation, treatment and follow up.
• Close physical contact (e.g. sleeping in the same room or nursing) with at-risk individuals (children under 5 years age, immunosuppressed adults, elderly, chronic ill health)
• Allergy to penicillin/amoxicillin
• Acute illness
  ○ Current illness
  ○ Acute illness within 3 days prior to inoculation
  ○ Antibiotic treatment within 2 weeks of inoculation
• Chronic illness that may impair immune response or impair ability to comply with study procedures and safety monitoring (e.g. HIV, diabetes).
• Taking immunosuppressive medication that may include but is not limited to steroids and steroid nasal spray.
• Pregnancy - minimise risk of pneumococcal disease
• Involved in another clinical trial unless observational or in follow-up (non-interventional) phase
• History of drug or alcohol abuse. This is very difficult to ascertain in a history therefore we will exclude people reporting drinking alcohol more than twice per week.
• History of smoking
  ○ Current regular smoker (smokes daily/ smokes > 5 cigarettes per week) - minimise risk of pneumococcal disease
  ○ Recent smoker, i.e. within the last 6 months - minimise risk of pneumococcal disease
  ○ Ex-smoker with a significant smoking history (>10 pack years) – minimise risk of pneumococcal disease
• Unable to give informed consent
• In case of any uncertainty or concern, the principal investigator (PI) will take clinical responsibility for the decision.
• Participant is positive for *S. pneumoniae* serotype 6B on nasal wash sample at visit C. Current natural carriage of serotype 6B would preclude determination of our primary outcome.

### Screening and preliminary assessment

Following consent, inclusion and exclusion criteria, data and samples will be collected.

• **Initial clinical examination** will include a focused clinical history and targeted clinical examination involving auscultation of the lung fields and heart sounds. Participants will be informed if their clinical examination is normal. Should a previously unrecognised abnormality be identified, this will be explained to the individual, and appropriate investigations and follow-up will then be arranged by the study team. Further participation will be determined at the discretion of the study doctor dependent on the nature of the abnormality detected. If the participant is not eligible due to an acute illness, they may be re-screened at a later date with their verbal consent.

• **Blood samples** will be obtained using venepuncture by an appropriately trained team member. A maximum of 100mL of blood will be collected in each phase of the study (total 200mL if participant consents to re-challenge after one year) for analysis including a full blood count. The volume of blood collected for each laboratory test is detailed in Table 1. Participants with an abnormal full blood count will be excluded from the study as determined by the clinical team.

• **Nasosorption** will be obtained before the nasal wash. The strips are similar to blotting paper and developed by Hunts Development Ltd (UK). Strips collect concentrated nasal lining fluid before the nasal wash to measure inflammatory responses induced by infection that may be associated with increased colonisation density and acquisition. Concentrated nasal fluid will be used to measure cytokine levels by multiplex bead array. Blotting paper will be held inside the nostril for up to 3 minutes until soaked. These will then be removed and placed in a microcentrifuge tube for storage.

• **Nasal wash** will be performed using an established method ^18^. This is a well-used and validated technique to collect nasal bacterial specimens used successfully during our feasibility study. Briefly, 5ml of sterile saline is instilled and held for a few seconds in the nares before being expelled into a sterile Galli pot. This is repeated twice in each naris using 20ml saline in total. In the event of nasal wash loss (defined as cough/sneeze/swallow), the procedure may then be repeated to obtain an adequate specimen. Should the nasal wash reveal natural colonisation of pneumococcus, the participant will continue in the study and receive the inoculation as per protocol, unless the natural serotype is identified as 6B, in which case the participant will be excluded from the study prior to inoculation (primary outcome cannot be determined). All pneumococcal carriage negative participants will continue in the study.

• **Nasal cells** will be collected using a nano-sampling method in which cells are obtained through minimally invasive superficial nasal scrape biopsies (Rhino-Pro Nasal Curette, Morphew Curettes). Experience from our feasibility study ^7^ demonstrates that participants can be biopsied multiple times with no significant side effects. Nasal cell samples will be obtained after nasal wash samples have been taken. Up to 4 samples (2 per nostril) will be obtained at each nasal sampling visit. If the researcher finds that the sample is insufficient, for example no cells are visible on the rhinoprobe, the sample can be repeated immediately. The rhinoprobes are placed in transport media immediately after collection.

• **Throat swabs** will be performed by simple posterior pharyngeal swabbing on a dry swab and placed into culture media. This test will be used to determine infection with SARS-CoV-2 by viral PCR prior to inoculation as part of screening and stored for batch classical microbiological testing (data not used for screening).

• **Saliva** will be collected by a salivette held for saturation or a spit in the tube.

• **Urine sample** will be taken for a pregnancy test as part of the safety screening. 

### Pneumococcal inoculation

***Preparation of bacterial stock and inoculation***

• **Initial preparation and transfer to MLW:** Microbiological and sequence confirmation of the purity of the inoculum will take place at LSTM and reference Public Health England laboratories prior to the onset of the study. Once complete, the microbiologically and genetically confirmed *S. pneumoniae* capsular serotype 6B strain BHN418 will be transferred from the LSTM to MLW. Microbiological and serotype confirmation will take place at MLW upon receipt.

• **Preparation of bacteria for carriage studies at MLW:** LSTM will provide MLW with mid-log broth cultures of pneumococcus, frozen at -80°C in aliquots of glycerol-enriched media. Immediately prior to participant inoculation, aliquots will be thawed, washed twice, and re-suspended in sterile saline at an appropriate density for each inoculation dose. Our validated Standard Operating Procedure (SOP), based on LSTM protocols, will be used.

• **Confirmation of accuracy:** Following culture by the laboratory team at MLW, aliquots of the bacterium will be returned to the UK, as a quality control measure, to confirm the identity, purity and penicillin sensitivity of the isolate. These bacterial isolates will be sequenced in the UK to confirm identification.

• **Regulatory approval:** *S. pneumoniae* transfer will be reported to the Malawian Pharmacy, Medicines and Poisons Board (PMPB, national regulatory authority) prior to initiation of the study. For our feasibility study, PMPB confirmed through the NHSRC that Malawi would adopt the UK Medicines and Health care products Regulatory Authority (MHRA) position that controlled human infection inoculation with pneumococcus is not an investigational medicinal product. However, the MHRA does give any challenge agent due consideration if used as part of a vaccine study (in which the vaccine is an IMP). PCV13 is an investigational medicinal product and has been approved by the Malawi Pharmacy and Medicines Regulatory Authority (PMRA: 10062020121) for this trial.

• **Inoculation:** Using a P200 micropipette, 0.1ml saline containing the desired dose of pneumococcus will be instilled into the nose. The participant will be seated in a semi-recumbent position. After inoculation, the participant will remain in this position for up to 15 mins. They will be given a post-inoculation advice sheet (including emergency contact details), thermometer, a course of amoxicillin and a daily symptom log to complete.

• **Dose escalation:** We will adopt a stepwise approach to escalating the inoculation dose, completing challenge in each dosage group before progression: 20,000 (n=40) → 80,000 (n=140) → 160,000 (n=20). We previously demonstrated successful carriage and safety at the 20,000 and 80,000 doses in our feasibility study ^7– 9^ and the 160,000 has been safely used in the Liverpool with no adverse events ^12^.

### Randomisation

Participants will be individually randomized to either PCV-13 (Prevenar) or normal (0.9%) saline vaccination prior to primary inoculation. A random number generator will be used to generate the randomization sequence and randomization occurs using block randomization with random block sizes of 6, 8 and 10 to ensure balanced vaccine allocations. An independent statistician will run the randomisation code for study group allocations and allocations will be stored on a secure electronic portal at MLW. The research team will be blinded to the vaccination group. Procedure: The study pharmacist will be fully unblinded and prepare each PCV-13 or normal saline according to the randomisation code. Individual doses will be prepared to maintain research team and participant blinding. Individual doses will be identified only by a randomisation identification number and barcode. Participants will be randomised on an electronic tablet immediately after screening is completed and they are confirmed eligible. The tablet will hold the randomisation identification number and barcode details. Subsequently, the vaccine barcode will be electronically confirmed against the participant identification number using the electronic tablet prior to administration. Unblinding will occur once microbiological reporting is completed for the final participant at visit G (14 days after primary inoculation). Participants entering the second stage (visit H) of the study will therefore be unblinded.

### Vaccines

The vaccines will be purchased by the MLW clinical trials pharmaceutical team. The vaccines will be stored at the appropriate temperature and administered as per manufacturer’s instructions. Prevenar-13 will be purchased from Pfizer Pharmaceuticals and administered in a single dose via intramuscular injection (0.5mL supplied in a prefilled syringe). The normal (0.9%) saline will be administered as a single dose (0.5mL) by intramuscular injection. Our previous study in the UK used hepatitis A as the control vaccine ^6^, however this product is not licensed in Malawi such that saline was chosen for this study. The vaccines will be administered by experienced, trained health care professionals from MLW. Participants will be observed for 20–30 minutes following vaccination to ensure that they do not experience a reaction to the vaccine. If any adverse reaction occurs the volunteer will be transferred to the Queen Elizabeth Central Hospital High Dependency and Respiratory Unit (HDRU) or the Adult Emergency and Trauma Unit (if HDRU capacity is limited) and the study team informed. Immediate un-blinding will occur in the event of an adverse event requiring medical attention for which knowledge of the vaccine given will affect treatment and ongoing immediate care.

Normal saline has been chosen as a suitable control due to its long-established safety profile in vaccine trials and lack of effect on nasal colonisation/immunity. Sterile 0.9% saline vaccination is commonly used as a control for vaccine trials including in sub–Saharan Africa ^19^ and in a recently published COVID vaccine study ^20^. Sterile saline is licensed for use for dissolving drugs for IV, IM and SC injection and used commonly in clinical practice. Whilst injection with saline will not confer any benefit, participants will be offered PCV13 vaccination after unblinding and completion of the day 14 sample following final inoculation in phase 2 (week 56). PCV13 will also be offered to participants who do not take part in the second phase of the study (decline consent or ineligible after re-screening). 

### Determination of colonisation

Colonisation will be defined by the microbiology result of nasal washes taken at 2-, 7- and 14-days post inoculation. Nasal washes will be plated on to culture media and incubated overnight at 37°C in 5% carbon dioxide (CO _2_). Colonies will be confirmed as *S. pneumoniae* using classical microbiological techniques including (i) typical draughtsman-like colony morphology, (ii) the presence of α-haemolysis, (iii) optochin sensitivity and (iv) bile solubility. Typing by latex agglutination will be done using a commercial kit to confirm pneumococcal serogroup. Isolates will be frozen at -80 ^o^C for storage. Results from the cultured nasal wash will also be confirmed using PCR based methods of bacterial detection.

Monitoring of colonisation will be performed by microbiology analysis of nasal washes. This will include measurement of pneumococcal density by LytA PCR. Monitoring for COVID-19 co-infection will be performed by RT-qPCR analysis of throat swab samples

### Safety during colonisation

A three-day course of amoxicillin and a digital thermometer will be given on the day of inoculation. These will be carried by the participants for immediate treatment after phone consultation should the participant develop moderate or severe symptoms as described on the participant safety advice leaflet. Participants will be required to make text message/phone contact with a specified member of the research team before 1200 hrs every day for 7 days post-inoculation *regardless of whether they have symptoms or not*. Should they not make contact by the specified time, a member of the research team will contact the participant. If no contact is made, then a ‘secondary contact’ (established during screening visit) will be telephoned. During the post-inoculation period, participants will have access to a 24/7 on-call telephone service until the end of each phase (H and L) of the study.

### Termination of carriage

All study participants who are nasally colonised with pneumococcus serotype 6B at the end of the study will be asked to take oral amoxicillin 500mg three times daily for 3 days. This dose is based on our UK experience where there have been no attributable SAEs for CHIM in >1500 participants completing the programme This will be communicated at visit G, or by telephone in the unlikely event of only visit G samples (day 14 – see Table 1) showing carriage.

### Immunological assays

• **Antibody titres:** measurement of pneumococcal-specific protein and polysaccharide antibodies will be conducted on nasal wash, salivary and serum samples using ELISA as previously reported ^21^. This will allow us to identify potential antibody correlates of protection against pneumococcal colonisation.

• **Cellular responses:** measurement of pneumococcal-specific memory B and T cells will be performed on peripheral blood and nasal samples using flow cytometry and ELISPOT as previously reported ^22, 23^. This will allow us to identify potential cellular correlates of protection against pneumococcal colonisation. 

• **Cytokine profiles:** measurement of cytokines will be done on nasosorption and serum samples using multiplex bead array as previously reported ^24^. This will enable us to identify potential soluble marker correlates of protection against pneumococcal colonisation.

• **Host transcriptomic analysis:** measurement of host nasal mucosa transcriptomic profile will be done using RNA sequencing (RNAseq) on nasal cells and peripheral blood as previously reported ^22^. This will allow us to characterise potential nasal or blood transcriptomic signatures associated with protection or susceptibility to pneumococcal carriage.

• **Microbiome profiles:** characterisation of microbial communities will be done on throat swabs and nasal washes using next-generation sequencing and metagenomic analysis as previously reported ^24^.

Assays will be harmonised with LSTM standard operating procedures where possible to facilitate comparability between CHIM studies

### Sample size justification

The primary endpoint is the occurrence of pneumococcal colonisation determined by the presence of pneumococcus in nasal wash samples at any time point post inoculation detected using classical microbiology. Secondary endpoints will include density and duration of pneumococcal carriage in nasal wash.

Based on the pneumococcal dose of 80,000 cfu/naris and using data from our feasibility study ^7, 8^ and Liverpool-based studies ^6^, we estimate that 60% of the control (normal saline) group will be colonised with pneumococci following inoculation and 36% of the PCV-13 group, this equates to a 40% reduction in colonisation. We observed a 78% reduction in the UK based study ^6^. However, as Malawian data demonstrates decreased efficacy for PCV-13 in carriage reduction ^10^, we have powered the study at approximately half of this effect size. Based on these parameters, we will randomise 67 participants per arm for 80% at a 5% significance level (see Table 2). To correct for any potential drop out, we will randomise 70 participants to each arm (total 140 participants for primary outcome).

**Table 2.** Power calculations for PCV13 study.

Number per group	Carriage rate control	Carriage rate PCV-13 vaccine	PCV-13 carriage rate reduction	Power
**67**	0.6	0.45	0.25	0.41
**67**	0.6	0.42	0.30	0.55
**67**	0.6	0.39	0.35	0.69
**67**	0.6	0.36	0.40	0.80
**67**	0.6	0.33	0.45	0.89
**67**	0.6	0.30	0.50	0.95

An additional 40 participants will be randomised (1:1, PCV13 vs. normal saline) at the 20,000 pneumococcal dose. A further 20 participants will be randomised (1:1, PCV13 vs. normal saline) at the 160,000 pneumococcal challenge dose. These doses will be used to explore the effects of differential pneumococcal exposure on PCV-13 vaccine efficacy. Recent methodological developments in vaccine efficacy estimation highlight important gains derived from the use of multiple-dose over single-dose experimental designs ^25^. By extending our core experiment with smaller groups of participants at doses below and above 80,000 CFU, we will be in a position to not only apply the new methods to a human system for the first time, but also test their performance against a previously established mathematical model ^25^. Participant consent is required before screening procedures can be conducted. Any participant who consents but is subsequently determined to be ineligible will be replaced so that a total of 200 participants will be inoculated with *Streptococcus pneumoniae* for the first phase of the study. Important secondary endpoints including the immune responses, in particular humoral and cellular responses, will also be assessed. Immunological parameters will be compared between pre-vaccination and pre-inoculation values in paired analyses using parametric or non-parametric tests as appropriate.

### Analysis plan

Phase one and phase two of this study will be analysed separately. The reporting of this study will be prepared in accordance to the CONSORT 2010 ^26^ guidelines. A CONSORT diagram will summarise participant screening, enrolment, randomisation, inoculation, withdrawals, follow-ups and analysis. All continuous data variables will be summarized using the following descriptive statistics: N (size of relevant analysis population), n (size of analysis population without missing values), mean, standard deviation, median, 25 ^th^ percentile value, 75 ^th^ percentile value and interquartile range, minimum and maximum. The proportion of observed levels will be reported for all binary and categorical measures. When appropriate, corresponding exact, binomial 95% confidence intervals for proportions will be included.

The primary endpoint is the occurrence of pneumococcal colonisation determined by the presence of pneumococcus in nasal wash samples at any time point post inoculation detected using classical microbiology. Secondary endpoints will include density and duration of pneumococcus in nasal wash. While 140 participants will be randomised (1:1, PCV13 vs. normal saline) at the 80,000 CFU pneumococcal dose, an additional 40 participants will be randomised (1:1, PCV13 vs. normal saline) at the 20,000 pneumococcal dose and a further 20 participants will be randomised (1:1, PCV13 vs. normal saline) at the 160,000 pneumococcal challenge dose.

The primary endpoint will be analysed using generalised linear models (GLMs; log-binomial or, in case of convergence issues, logistic regression) with treatment as a single predictor, generating risk ratios and odds ratios together with their 95% confidence intervals of being colonized with pneumococcus between the PCV13 and normal saline control groups. We will use longitudinal data analysis methods, specifically generalised linear mixed models (GLMM) or generalised estimating equations (GEE), to analysis pneumococcal carriage status and density at individual time points with treatment, time and interaction between time and treatment as fixed effects and study participant as random effect / clustering variable.

Immunological parameters will be compared between PCV13 and placebo recipients, as well as between carriers and non-carriers using similar methods (GLMs for comparing parameters according to overall carriage status and/or treatment, GLMMS/GEEs for longitudinal analyses). The area under the curve (AUC) of pneumococcal colonization at days 2, 7 and 14 post inoculation will be calculated using the trapezoidal rule and the AUC will be analysed using a GLM with a single factor for treatment.

The effects of differential pneumococcal exposure on PCV-13 vaccine efficacy will be analysed using recent methodology developed by our team ^25^. Expanding vaccine efficacy estimation with dynamic models fitted to cross-sectional prevalence data post-licensure. All quantitative analyses will be conducted in the intention-to-treat population. Analyses will be conducted using the R environment for statistical programming and computing (v4.0.0 or higher). All computer code will be made publicly available on GitHub under a CC BY 4.0 license.

### Social science analytical plan

The social science evaluation requires qualitative data to understand volunteer perspectives of benefits, challenges, tolerability of study procedures, and any potential unintended effects through the study. All participants will be asked to complete an exit questionnaire upon completion of visit G (and visit L if consented for the second phase of the study) ( *Extended data* ^27^). This questionnaire will be completed by the participant on a tablet computer. Any participant who expresses concern(s) or dissatisfaction will be offered the opportunity to discuss in more detail as part of a semi-structured exit interview conducted as a separate appointment.

In the event that there are several structured exit interviews, all data collected will be audio-recorded and transferred to a qualitative data analysis software package (NVIVO), to enable analysis. Data will be analysed following broad deductively defined themes and inductively derived sub-themes. We will employ a combination of thematic coding in NVivo and use of frameworks to compare perspectives between different stakeholders (participants and study team members). Two researchers will undertake initial coding of an initial small number of transcripts, and then discuss and agree themes for further coding in the event that there are any further transcripts. Analysis will be on-going during fieldwork, using an iterative approach to identify emerging themes that can be clarified or explored further through later data collection. Data will be triangulated between methods and participant groups to cross-check information and provide a more comprehensive analysis. At this point, given responses in the feasibility study, we consider it unlikely that there will be a significant volume of exit interview material to analyse.

### Safety considerations and assessment

***Study design to ensure safety.*** Safety is paramount in human infection studies. While the risk to individuals of developing any infection is very low (40% Malawian adults experience natural colonisation at any time, and the incidence of invasive disease is 20/100 000 patient years ^28^), the study is designed to ensure any risk is minimal by appropriate:

• Study team selection and rigorous training in human challenge procedures
• Study design with staggered recruitment approach (sample size and dose)
• Careful serotype selection, demonstration of safety and acceptability in our feasibility study and 10 years of pneumococcal controlled human infection experience at LSTM
• Participant selection and exclusion criteria as detailed
  ○ Including exclusion of COVID-19 positive participants at screening visit and continued monitoring for infection at day 2, 7 and 14 after inoculation
• Participant education through a participant information sheet (provided)
• Rigorous safety procedures including daily monitoring and providing participants with a thermometer and a course of amoxicillin tablets in case of emergency
• 24-hour emergency telephone contact with researchers, including close individual daily monitoring, and access to hospital facilities and prompt treatment if required
• To further mitigate risk, participants will be provided with accommodation for three nights immediately following nasal challenge. If a participant determines that it is not possible to stay at study accommodation for three nights then a pragmatic approach to further study procedures will be taken. If the participant can demonstrate that they remain readily available by phone and accessible with transport, this need not affect the procedure. Twice daily checks could replace the resident period. In the Liverpool CHIM there is no resident period at all. If, however, the participant absconds or is intending to be uncontactable, then participation in the study will be stopped and antibiotics offered as at the end of the protocol.

We will schedule inoculations such that visit G is completed for each pneumococcal dose group (20,000 and 80,000) before progression to the next group (80,000 and 160,000 respectively).

The research investigators have successfully worked together to safely deliver our pneumococcal CHIM feasibility study in Malawi based on knowledge and experience from the Liverpool studies ^7^. No episodes of pneumococcal infection or serious unexpected serious adverse reactions (SUSARS) have occurred in any of our participants whether they had no carriage, natural carriage or experimental carriage.

***Vaccine safety.*** Prevenar-13 is a safe vaccine with a very low risk of adverse events ^29, 30^. It is currently licensed for use in children and in Malawi as part of the Childhood Immunisation programme (effective from 2011). In adults over 50 years of age vaccinated in US clinical trials the most commonly reported side effects to Prevenar-13 vaccination included: injection site pain/swelling/tenderness, fatigue, headache, muscle pain, limitation of arms movement, decreased appetite, chills and rash.

Normal (0.9%) saline is commonly used for control vaccination in vaccine efficacy trials. Normal saline is licensed for IV, IM and SC injection and is safe with minimal side effects ^20^.

Participants will be observed by trained research staff in the research clinic rooms after vaccination for a period of 20–30 minutes. This procedure is to mitigate the very low risk of any immediate drug reaction. The 3A research clinic rooms are situated directly opposite to a respiratory high dependency unit at Queen Elizabeth Central Hospital. In the unlikely event that a participant became unwell after vaccination, they would be transported to this ward for treatment and close monitoring.

***Participant safety procedures.*** We will provide all participants with a safety information sheet ^31^. This sheet classifies symptoms into mild, moderate and severe. We will take a proactive approach for participants, with daily contact up until day seven post inoculation and enquiry about development of any symptoms. We will also encourage participants to report development of any symptoms with 24-hour medical cover throughout the study visit schedule. In the event of illness, we will apply standardised operating safety procedures for participants. The research team will pay for any costs associated with these procedures and participants will incur no out of pocket expenses.

***Mild illness.*** Mild illness is defined as development of any new symptom that is of concern to the participant during the study period. These may include mild coryzal symptoms such as blocked or runny nose. Participants will be encouraged to contact the research team by telephone within office hours. During the telephone assessment participants will be screened for potentially concerning moderate and severe symptoms. If there is any concern, participants will be invited to the research clinic for a detailed clinical assessment by one of the study doctors. The assessment will be fully documented within study records including rationale for any treatments and/or required modifications to study procedures.

***Moderate Illness.*** Moderate illness events are defined as fever with temperature >37.5°C, shivering, headache, new rash, drowsiness, cough, new earache and or new eye infection. Participants will be clearly instructed to call the research team at any time of the day upon development of any of these symptoms. During the telephone assessment participants will be screened for severe symptoms and requested to attend either the research clinic (office hours) or Mwaiwathu hospital (outside of office hours) for medical assessment. If severe symptoms are identified on telephone assessment, participants will be asked to take antibiotics from their supplied emergency pack and attend Mwaiwathu hospital. Study doctors will assess the participants at either the research clinic or Mwaiwathu and offer appropriate treatments. The assessment will be fully documented within study records including rationale for any treatments.

***Severe illness.*** Severe illness events are defined as any symptoms that cause serious concern to participants following inoculation. We have purposefully taken a conservative approach to encourage participants to seek early medical advice and treatment if they are concerned. Participants will be counselled as to what symptoms the study team may be particularly concerned about during the consent process. These would include any potential symptoms of sepsis. In the event of severe illness, participants are requested to start taking the provided emergency antibiotics, directly attend Mwaiwathu hospital and contact the study team. A study doctor will attend the patient in Mwaiwathu and offer appropriate investigations (may include blood culture if clinically indicated) treatments. The assessment will be fully documented within study records including rationale for any treatments.

***COVID-19 specific participant safety procedures.*** Participants who screen positive for COVID-19 will be excluded from the study. The study team will offer standardised advice to such participants including self-isolation measures to reduce onward transmission in line with Malawi Ministry of Health guidance ^32^. After an initial negative test, we will continue to screen participants for development of COVID-19 after pneumococcal inoculation at visits 4, 6 and 7. No additional samples will be required, we will use throat swabs to test for COVID-19 using RT-qPCR in line with CDC guidance ^33^. Participants will be notified if they become positive for COVID-19 during the study and excluded from further visits. This procedure is to facilitate self-isolation in line with guidance and to reduce the risk of transmission. If the participant has concurrent pneumococcal carriage, they will be advised to commence antibiotics to reduce the theoretical risk of bacterial co-infection. We will replace participants who are excluded during post-inoculation follow up to recruit the pre-specified allocated number of patients to each group.

***Evaluation of adverse events and serious adverse events***

**Adverse events:** Non-serious adverse events (AEs) will be collected systematically during the research and recorded in the case report form. Participants will keep a log of symptoms and this will be summarised and reported to the Data, Safety and Monitoring Board (DSMB). 

**Serious adverse events:** Any *serious (*AE *),* as defined in ICH-GCP occurring to a research participant, will be reported to the DSMB, NHSRC and study sponsor within 24 hours of the study team becoming aware of the event. In this event, the research will be stopped temporarily for investigation and any further work deferred until DSMB and NHSRC advice has been provided to the Trial Steering Committee (TSC) for consideration. 

**Risks to researchers:** These are standard clinical (needle stick) and laboratory (biohazard) risks. Experienced staff will carry out procedures that are within their competencies in accordance with standard operating procedures regulated by good clinical practice and national guidelines. Appropriate risk assessments are in place for all laboratory SOPs. All laboratory work will be conducted in an appropriately rated laboratory in line with health and safety regulations for research with human tissues / infectious agents. All staff will be provided with and training in the appropriate use of personal protective equipment to reduce the risk of COVID-19 infection. We will use UK Public Health England PPE guidance recommendations ^34^.

***The Data Safety Monitoring Board (DSMB).*** The DSMB will be established prior to commencing this study and include members expert in controlled human infection, safety and statistical procedures. Prof Rob Read will Chair - Prof Read has chaired the LSTM DSMB for 10 years.

The DSMB will monitor the study and advise the TSC, including the PI and study team. Briefly, all SAEs will be reported to the DSMB and study sponsor within 24 hours and recruitment/inoculation paused pending DSMB review and recommendation to the TSC in line with GCP guidelines. The study team will provide at least monthly update (by email) on all recruitment to the DSMB. The DSMB will meet formally (by telephone conference) biannually and in the event of any SUSARs.

***Stopping criteria.*** This study will pause in the event of a SAE or on completion of the Protocol. Thereafter, DSMB and NHSRC members will assess the event before advising the Trial Steering Committee on measures required to resume the study or if the study should be stopped. The study will only be resumed if both the DSMB and NHSRC are satisfied that required measures are in place. If the DSMB or NHSRC recommends the early stopping of the study, all reasons must be stated clearly. If there are DSMB or NHSRC members with dissenting views, these must be appended to the recommendations. If there are no trial related serious adverse events, the study will stop upon completion of the protocol.

### Ethical considerations

***Autonomy.*** The participants will be given high-quality information that is written and spoken using common lay terms without jargon to allow them to understand the research objectives and the risks and benefits of all procedures. They will then be given time to consider the information before consenting to any involvement. At no stage should the participant feel pressured or persuaded into participating in the research study.

Participants have the right to withdraw their consent and therefore withdraw from the study at any time without giving reason. If a participant withdraws from the study, we will recruit an alternative participant to complete the study.

***Non-maleficence.*** Inoculation of *S. pneumoniae* will be as per the established and safe protocols and will be performed by highly trained staff with close supervision (24hr on call access to medical professionals involved in the study) and follow-up. Specific inclusion and exclusion criteria are set to further protect the participant. Experience and trained research staff will perform venepuncture and nasal sampling.

***Beneficence.*** Participants will receive a health check including HIV-infection status assessment when taking part in this study. Participants also have the potential to benefit from completed vaccine schedules during the study. Patients randomised to normal saline will be offered PCV-13 upon completion of study related activities at visit L. In addition, participants may benefit from a better understanding of clinical research, they may also benefit from a sense of contributing to medical research in a valuable way. Participants will be remunerated for their time and inconvenience – discussed below.

***Justice.*** This must be balanced with non-maleficence. Inclusion and exclusion criteria are in place primarily to protect individuals from undue risk.

***Ethical approvals.*** Ethical approvals for this study have been granted in country by the Malawi National Health Sciences Research Committee (NHSRC, REF: 16/07/2519) and from a Liverpool School of Tropical Medicine (LSTM) institutional perspective (REF: 20-021).

***Trial registry.*** This trial is registered with the Pan African Clinical Trial Registry (PACTR, REF: PACTR202008503507113).

***Regulatory approval.*** The trial has been approved by the Malawi Pharmacy and Medicines Regulatory Authority (PMRA/CTRC/III/10062020121)

### Remuneration

It is intended that financial factors will not significantly influence an individual's decision to participate in this study. We reimburse participants for out-of-pocket expenses such as travel, and compensate time spent and burden. The sums offered in this study are consistent with remuneration guidelines published in Malawi, paid pro-rata (per activity and not dependent on study completion) ^35^.

If a participant withdraws from the study early, they will be compensated for the parts they took part in up until the time they withdrew. Payments are summarised in Table 3 and Table 4, MK 11,000 will be paid at the end of each study visit for Phase 1. If the volunteer consents to participate in Phase 2, MK 10,000 will be paid at the end of each study visit. For volunteers who are approached and agree to be interviewed for social science activities at the end of visit G and visit L there will be an additional MK 7000 offered to compensate for their time and transportation.

**Table 3.** Research participant remuneration for Phase 1 (visits A-G). *Accommodation and board costs will be paid directly by the research team. Participants will not receive compensation to attend the information visit (vist A). Visit D will cause mild discomfort. Visits B, C, E, F and G will cause mild/moderate discomfort.

MARVELS Research Participant Remuneration (based on Malawian guidelines ^35^)			
Reimburse expenses	Rate in MK	Number of events	Total
a) Transport	900	7	6,300
b) Subsistence (one meal)	1500	7	10,500
c) Accommodation (one night) *	15000	3	0
**Compensation**			
Total time travelling (hrs)		7	
Total time in research facility (hrs)		21 research 72 accommodation	
Time in days (day = 8 hours)	1000	12.5	12,500
Procedure A (mild discomfort)	2000	1	2,000
Procedure B (moderate discomfort)	6000	5	30,000
Procedure C (long or complex)	10000		
**TOTAL for study** **(Phase 1)**			65,800
**AVERAGE per visit**			11,000

**Table 4.** Research participant remuneration for Phase 2 (visits H-L). *Accommodation and board costs will be paid directly by the research team. Visit I will cause mild discomfort. Visits H, J, K, and L will cause mild/moderate discomfort.

MARVELS Research Participant Remuneration (based on Malawian guidelines ^35^)			
Reimburse expenses	Rate in MK	Number of events	Total
a) Transport	900	5	4,500
b) Subsistence (one meal)	1500	5	7,500
c) Accommodation (one night) *	15000	3	0
**Compensation**			
Total time travelling (hrs)		5	
Total time in research facility (hrs)		15 research 72 accommodation	
Time in days (day = 8 hours)	1000	11.5	11,500
Procedure A (mild discomfort)	2000	1	2,000
Procedure B (moderate discomfort)	6000	4	24,000
Procedure C (long or complex)	10000		
**TOTAL for study (Phase 2)**			49,500
**AVERAGE per visit**			10,000

### Confidentiality and anonymity

Only authorised members of the research team will have access to any personal information. Only information of direct relevance to the study will be collected ( *Extended data* ^17^). All electronic records containing personal information will be stored in a password protected database on a password protected server at MLW. Electronic case report form data will be collected on encrypted study-specific tablet devices and synchronised daily onto the MLW server. This approach is well established at MLW, and the policy governing MLW data management policy is available upon request. Paper documentation containing personal information will be kept in a locked filing cabinet in a locked room in the Queen Elizabeth Central Hospital research clinic.

Each participant will be assigned a unique non-identifiable study number by a member of the clinical research team at recruitment. Unlinked non-identifiable clinical data will be stored and analysed at the MLW-laboratories and collaborating laboratories.

For any exit interviews carried out, the following ethical considerations will be applied to ensure privacy and confidentiality is upheld. Following each interview, audio files will be transcribed verbatim into written Chichewa or English (depending on language used in interview) and translated to English where necessary. The research assistant responsible will save the audio file on a secure cloud-based network drive that will only be accessible to the social science team of researchers. Any paper copies will be stored in a locked filing cabinet.

Participant names will not be included in transcripts or file names; instead, participants will be given ID numbers. Enrolment logs and any consent documents that include participant names or contact details will be stored separately in a locked filing cabinet. Electronic recordings and transcripts will be stored on a secure cloud-based drive for a minimum of five years from the end of data collection, to allow data analysis, publication, and ability to the wider scientific community to check our results.

### Samples and data

***Sample and data storage.*** MLW will act as custodian for all data and samples collected during the study. Consent will be obtained from the participant to use the samples for this research only. Samples will be stored for a maximum of five years. 

***Collaborating laboratories.*** Samples will also be sent to national and international collaborating laboratories to utilise specialist expertise not available in Malawi. Samples will be labelled with the anonymised study number. Consent will specifically be obtained from the participants to allow samples to be sent to our collaborators.

### Social science

All participants will be asked to complete a digital questionnaire upon completion of study related activities at the end of visit G and visit L (see Table 1). If a participant expresses any concerns or dissatisfaction, they will be offered a follow up exit interview at an additional visit to explore the issue(s) in more detailed with a trained qualitative researcher. The interview will take place on a separate date and location and with a qualitative researcher who has not been involved in clinical delivery of the study. The interview will be semi-structured and focused on concerns raised by the participant, lasting approximately 30minutes. Participants will be offered additional compensation for time and travel to this interview.

## Dissemination of findings

The findings from this study will be disseminated amongst the scientific community. We intend to publish our findings in peer reviewed scientific journals and present data at appropriate local, national and international conferences. We will produce a close-out report for the NHSRC at the end of the study and a final report once data are published. In addition, we will produce a lay report of our findings which will be made available to all participants.

## Sponsorship and indemnity

**Sponsorship:** The Liverpool School of Tropical Medicine (LSTM) is the sponsor (REF: 20-021).

**Indemnity:** We have purchased no fault clinical trials insurance to cover research participants for this study. This has received regulatory endorsement by the Malawi National Commission for Science and Technology (NCST, REF: NCST/RTT/2/6).

## Future development of the MARVELS programme

Success in this project will result in:

A)    Measurement of the effect of PCV13 on EHPC. If the EHPC method shows a vaccine effect in Malawian adults, this will allow consideration of CHIM methods to predict the efficacy of novel vaccines in preventing pneumococcal carriage (and hence transmission) in Malawi.

B)    Determination of immunological mechanisms for observed differences in PCV-13 induced mucosal (nasal carriage) protection between UK and Malawian populations

C)    Determination of 12-month PCV-13 induced host protection against pneumococcal carriage

D)    A greater understanding of the potential protective effects of carriage acquisition against future pneumococcal challenge in the control group.

E)    In-country capacity building to conduct future vaccine testing studies

Future work will be planned to build on both of these anticipated outcomes by engaging with vaccine manufacturing companies and mucosal adjuvant programmes to develop more efficacious pneumococcal vaccines for the Malawian population.

## Study status

At time of manuscript submission (20 ^th^ August 2021), study recruitment has commenced with successful screening and randomisation of 40 participants. However, the study was paused due to increasing cases of SARS-CoV-2 within Malawi on the 16 ^th^ June 2021.

Future amendments to the protocol will require approval by the National Health Research Ethics Committee and the Sponsors.

## Discussion

This study will determine if vaccination with PCV13 is protective against pneumococcal carriage in healthy adult Malawian volunteers. We have designed the study such that findings will be exactly comparable with a previously completed trial in the United Kingdom ^6^. Expanding on this study, we will also determine how pneumococcal dose influences carriage in vaccinated participants and determine longer term protection against carriage 12 months after vaccination. Concurrently, we will investigate underpinning immunological mechanisms for disease susceptibility to inform the discovery and development of new vaccine candidates. Generation of these data are vital to understand observed differences in vaccine effectiveness in the sub-Saharan African context and to inform the discovery and development of new vaccine candidates.

## Data availability

### Underlying data

No data are associated with this article. Investigators wishing to collaborate and/or access data, samples and materials should contact the senior author (Gordon) directly.

### Extended data

Harvard Dataverse: EHPC Safety Leaflet. https://doi.org/10.7910/DVN/3LEAYH ^31^

Harvard Dataverse: MARVELS PCV13 Data Collection Forms. https://doi.org/10.7910/DVN/PUEH0X ^17^

This project contains the following extended data:

• Exit questionnaire
• Screening questions
• Visits question schedule

Harvard Dataverse: MARVELS PCV13 Participant information and consent forms. https://doi.org/10.7910/DVN/4PHN2H ^16^

This project contains the following extended data:

• Phase 1 Consent Form
• Phase 2 Consent Form
• Phase 1 Participant Information Leaflet
• Phase 2 Participant Information Leaflet

### Reporting guidelines

Havard Dataverse: SPIRIT checklist for ‘The influence of pneumococcal conjugate vaccine-13 on nasal colonisation in a controlled human infection model of pneumococcal carriage in Malawi: a double-blinded randomised controlled trial protocol’. https://doi.org/10.7910/DVN/1QAUYA ^27^

Data are available under the terms of the Creative Commons Zero "No rights reserved" data waiver (CC0 1.0 Public domain dedication).
