# Supplementary material for: The influence of pneumococcal conjugate vaccine-13 on nasal colonisation in a controlled human infection model of pneumococcal carriage in Malawi: a double-blinded randomised controlled trial protocol
Source: Wellcome Open Res. 2022 Jun 16;6:240. Originally published 2021 Sep 20. [Version 2] doi: 10.12688/wellcomeopenres.17172.2 (PMC9549377; doi:10.12688/wellcomeopenres.17172.2)
Supplement: Peer review history

# Peer review: Reviewer response for version 2

**DOI:** 10.21956/wellcomeopenres.19931.r51100

**Published:** 2022-06-23

**Authors:** Jaranit Kaewkwungal

**Affiliations:** 1 Department of Tropical Hygiene, Faculty of Tropical Medicine, Mahidol University, Bangkok, Thailand

The researchers have revised the manuscript accordingly. The manuscript is quite clear now.

Is the study design appropriate for the research question?

Yes

Is the rationale for, and objectives of, the study clearly described?

Yes

Are sufficient details of the methods provided to allow replication by others?

Partly

Are the datasets clearly presented in a useable and accessible format?

Not applicable

Reviewer Expertise:

Research methodology & statistics

I confirm that I have read this submission and believe that I have an appropriate level of expertise to confirm that it is of an acceptable scientific standard.

# Peer review: Reviewer response for version 2

**DOI:** 10.21956/wellcomeopenres.19931.r51099

**Published:** 2022-06-22

**Authors:** Vivak Parkash

**Affiliations:** 1 York Biomedical Research Institute, Hull York Medical School, University of York, York, UK

The authors have incorporated the suggestions made and I am now satisfied with the latest version of the manuscript.

Is the study design appropriate for the research question?

Yes

Is the rationale for, and objectives of, the study clearly described?

Yes

Are sufficient details of the methods provided to allow replication by others?

Yes

Are the datasets clearly presented in a useable and accessible format?

Yes

Reviewer Expertise:

Controlled human infection models, clinical research, clinical infectious diseases

I confirm that I have read this submission and believe that I have an appropriate level of expertise to confirm that it is of an acceptable scientific standard.

# Peer review: Reviewer response for version 1

**DOI:** 10.21956/wellcomeopenres.18971.r50417

**Published:** 2022-06-10

**Authors:** Jaranit Kaewkwungal

**Affiliations:** 1 Department of Tropical Hygiene, Faculty of Tropical Medicine, Mahidol University, Bangkok, Thailand

The study is interesting and well-prepared. There are some issues that should be elaborated as follow:

1. The study is planned as a double-blinded randomised controlled trial of adult healthy human participants experimentally exposed to escalating doses of *S.* *pneumoniae* in the nasopharynx. The escalating the inoculation dose: 20,000 (n=40) → 80,000 (n=140) → 160,000 (n=20). In the description of study workflow, however, it is not clear how the escalating steps will be performed. Will the study start with 20,000 then follow by 80,000 and 160,000, respectively? Will there be any criteria to stop escalating, or will there be any interim analysis to move forward? The sample size calculation was done for the main dose (80,000) of interest. In terms of data analysis, what kind of analytical procedures will be done for the escalating doses? Please provide more details on these aspects.
2. Regarding the sample size – how did you handle dropouts, those lost to follow-up/incomplete information/test results? Will there be replacement for the missing or incomplete cases? What could be the optimal sample size needed for Phase II, the re-challenging phase?
3. Is the calculated number of sample size enough to capture any AEs/SAEs? How AEs/SAEs be analysed?
4. Will there be any interim analysis between Phase I and Phase II?
5. Please describe more on what/how the social science data will be collected and analysed.

Is the study design appropriate for the research question?

Yes

Is the rationale for, and objectives of, the study clearly described?

Yes

Are sufficient details of the methods provided to allow replication by others?

Partly

Are the datasets clearly presented in a useable and accessible format?

Not applicable

Reviewer Expertise:

Research methodology & statistics

I confirm that I have read this submission and believe that I have an appropriate level of expertise to confirm that it is of an acceptable scientific standard, however I have significant reservations, as outlined above.

# Response

**Published:** 2022-06-11

**Authors:** Ben Morton

**Affiliations:** Liverpool School of Tropical Medicine, Malawi

**Dose escalation:**

 Dose escalation will be conducted in a stepwise manner. We have now clarified this in the relevant section. Criteria to step escalating will be based on participant safety governed by our data safety monitoring board and regulators (detailed in stopping criteria section). Our detailed statistical analysis plan will be finalised and published prior to database lock before analysis and will provide extensive detail on planned procedures.

**Dropouts:**

 All participant dropouts will be replaced, this includes any participants who miss a study visit. We have robust QA/QC procedures in place to reduce the risk of any incomplete information or test results for participants who attend follow up clinics. We did not conduct a power calculation for the secondary outcome measure in phase II of the study. Our aim is to re-recruit as many participants from phase 1 as possible.

**AEs/SAEs:**

 We will explicitly ask about AEs and SAEs at each study visit and report these in a similar fashion as our feasibility study ( https://www.thelancet.com/journals/ebiom/article/PIIS2352-3964(21)00372-8/fulltext).

**Interim analysis:**

 Yes, we plan to analyse phase 1 and phase 2 separately. We have now made this clearer in the manuscript analysis plan.

**Social science:**

 We had provided extensive details on how social science aspects will be collected analysed within the analytical plan but accept these could be highlighted further. We have now attempted to do this under a specific subheading.

# Peer review: Reviewer response for version 1

**DOI:** 10.21956/wellcomeopenres.18971.r50419

**Published:** 2022-06-10

**Authors:** Vivak Parkash

**Affiliations:** 1 York Biomedical Research Institute, Hull York Medical School, University of York, York, UK

Despite the availability of conjugate pneumococcal vaccines, pneumococcal respiratory (and other invasive) infections remain a global health concern. In the Malawian population, both nasal carriage and invasive infections are higher than UK populations suggesting decreased vaccination efficacy. The authors have described a protocol for a controlled human infection for pneumococcus (via nostril inoculation) in the context of measuring efficacy of a pneumococcal 13-valent conjugate vaccine. This study is 1:1 double-blinded RCT, with re-challenge at 1 year. Studies such as this are critical in improving the understanding of disease progression in distinct populations, as well as differences in effectiveness of treatments and vaccines.

 The authors must be commended on their efforts to translate an existing established CHIM from the UK to a Malawian setting in order to compare the effect of carriage in distinct populations. Preliminary work has already been undertaken to determine the feasibility of controlled pneumococcal nasal infection in a Malawian population. The study is well designed and constructed, with a wealth of underpinning studies covering public engagement, development of the CHIM in a UK population and established and validated method for delivering the challenge agent. The authors have made sincere efforts to ensure safety of participants throughout the study and the robustness of the protocol is clear from the outset. This protocol supplies sufficient information to allow others to replicate this work. I have included some minor comments below on various sections which the authors may find useful to increase the robustness of this work and improve the accuracy and readability.

 Introduction:

 A very brief description of the characterization and provenance of the challenge agent would be useful, including potential availability for other groups if appropriate.

 Ethical considerations:

 Although published extensively elsewhere, it would be useful to detail the key PPIE themes in brief here. This will help the reader understand the excellent engagement work that has been carried out, but also the public enthusiasm and concerns for such studies.

 Study protocol:

 Whilst the rationale for use of saline control has been given, please do ensure rational given for using saline vs another control vaccine (eg hepatitis A), especially as previous studies have employed hepatitis A control vaccine. Please do note that the information leaflet (extended data) states hepatitis A vaccination rather than saline will be used and this must be corrected.

 Primary inoculation:

 Although clarified later it, would be useful to state that this escalation is in relation to groups of patients rather than the same patient receiving an escalating dose.

 Secondary inoculation:

 Please give rationale for the dose given at re-challenge.

 In Table 1 you mention U&E in the legend, but not in the table itself. Presumably the serum is to be used for biochemistry? Or will it have further uses, i.e. serology?

 The visit questions are very comprehensive, however the investigators may wish to consider use of a diary card to capture additional symptomology that may fall between visits. The visit questions are phrased to capture current rather than past symptoms, and you may wish to consider re-phrasing these, or allowing for recording of symptoms that occur between visits. If daily phone calls will capture this information, then this must be highlighted (eg in Table 1). A daily diary card may not be suitable for this study but could be considered by the investigators in future if appropriate.

 The use of exit questions is commendable and will help improve future studies. The investigators may however wish to consider validated questionnaires that assess participant acceptability throughout the study (eg a quality of life questionnaire at the beginning, and then at exit, or other suitable questions). What would occur in the unlikely event that a participant did miss a visit, could they re-attend at the next possible juncture? Would they be excluded from the 2nd phase of the study (re-challenge)?

 Safety net advice could be considered between visit G and H.

 Exclusion criteria:

 The exclusion criteria is extremely robust and pragmatic. Do the investigators feel there is any value in taking and excluding on the basis of a viral throat swab (in addition to the planned SARS-CoV-2 swab) for routine viral respiratory infections given their potential impact on participants’ involvement? There is subsequent reference to throat swabs in the ‘Screening and preliminary assessment’ section, which makes reference to culture. It would be useful to clarify this i.e. bacterial culture, viral PCR, organisms screened for etc. The exclusion of people living with HIV has a clear basis in increased rates of pneumococcal disease. The investigators may wish to consider testing for HTLV in future studies if appropriate given some data suggesting increased rates in Malawi. Will anyone be excluded based on nasosorption?

 Regulatory approval:

 It would be worth clarifying the position that although the challenge agent is not an IMP, the MHRA should give any challenge agent due consideration if used as part of a vaccine study (in which the vaccine is an IMP).

 Termination of carriage:

 Although amoxicillin 500mg/3days is very likely to terminate infectious carriage, is there any evidence base or rationale for using this length of treatment that the authors could offer?

 If participants’ exhibit signs or symptoms of being systemically unwell, is there a possibility or allowance for blood cultures to be taken to determine bacteraemia? Although I appreciate that these individuals may be excluded from the study/unblinded and receive treatment, time to bacteraemia or simply evidence of bacteraemia may be a useful safety feature going forward.

 Immunological assays:

 It would be useful in terms of harmonisation, if feasible, that the authors list the panels/protocol etc. intended for use. This may allow some comparability with other CHIM studies.

 The investigators have made a valiant effort in their recruitment of participants which has unfortunately been paused as a result of the COVID-19 pandemic. I would be very interested in seeing how the study progresses.

Is the study design appropriate for the research question?

Yes

Is the rationale for, and objectives of, the study clearly described?

Yes

Are sufficient details of the methods provided to allow replication by others?

Yes

Are the datasets clearly presented in a useable and accessible format?

Yes

Reviewer Expertise:

Controlled human infection models, clinical research, clinical infectious diseases

I confirm that I have read this submission and believe that I have an appropriate level of expertise to confirm that it is of an acceptable scientific standard, however I have significant reservations, as outlined above.

# Response

**Published:** 2022-06-11

**Authors:** Ben Morton

**Affiliations:** Liverpool School of Tropical Medicine, Malawi

**Introduction**

 We have now included additional information on characterisation and provenance of the challenge agent in this section. We have also provided details on how to collaborate (access to data/samples/materials) in the data availability section.

**Ethical considerations**

 We have now provided additional detail in this section, detailing key themes.

**Study protocol**

 Further detail now provided in the vaccines section:

 “Our previous study in the UK used hepatitis A as the control vaccine ^6^, however this product is not licensed in Malawi such that saline was chosen for this study”

 We have now uploaded a more recent version of the consent form to the extended data. server with correct reference to Saline vaccination.

**Primary inoculation**

 Group rather than individual dose escalation has now been clarified.

**Secondary inoculation**

 Rationale now provided.

**Table 1**

 We have further clarified the table legend.

**Diary card**

 We have not used a daily diary card for this study. Participants contact the study team by text message daily after inoculation, documenting their temperature and if they have any symptoms. This is used as a trigger for further clinical enquiry if any issues are identified

**Participant acceptability**

 Following on from our published acceptability study ( https://wellcomeopenresearch.org/articles/6-89), we decided that an exit questionnaire was the most pragmatic option in this much larger study of 200 volunteers. We have retained the option to discuss participation in more detail through semi-structured interview within the protocol (see analysis plan) triggered by the exit questionnaire (e.g. if the participant raises any questions or concerns).

**Safety net advice**

 We certainly offer this and have now included reference in the protocol – see “participants, schedule and timelines”.

**Exclusion criteria**

 We use the throat swab for both bacterial and viral culture during the study with the throat swab specifically used for SARS-CoV-2 detection – we have now clarified this point. The suggestion on HTLV is noted, thank you. No participants will be excluded based on nasosorption.

**Regulatory approval**

 We have now corrected this section

**Termination of carriage**

 The recommendation to use 500mg amoxicillin TDS for three days is based on our UK experience of the 6B model. Whilst we cannot offer specific evidence for this treatment course in terms of continued colonisation, no attributable SAEs have occurred in >1500 participants using this dose in UK experience. If a participant feels unwell they are assessed by a study clinician and further clinical investigations are conducted (including blood culture if clinically indicated). We have now documented this in the “severe illness” section of the protocol.

**Immunological assays**

 We agree that harmonisation is important and have now added the following to the immunological assays section: “Assays will be harmonised with LSTM standard operating procedures where possible to facilitate comparability between CHIM studies”.
